# Monodisperse Manganese‐Vanadium‐Oxo Clusters with Extraordinary Lithium Storage

**DOI:** 10.1002/advs.202402616

**Published:** 2024-06-03

**Authors:** Wensi Tang, Tianyu Qiu, Zhiyuan Hu, Yingqi Li, Ruiqi Yao, Yonghui Wang, Xingyou Lang, Huaqiao Tan, Yangguang Li, Qing Jiang

**Affiliations:** ^1^ Key Laboratory of Polyoxometalate and Reticular Material Chemistry of Ministry of Education, Faculty of Chemistry Northeast Normal University Changchun Jilin 130024 China; ^2^ Key Laboratory of Automobile Materials (Jilin University), Ministry of Education and School of Materials Science and Engineering Jilin University Changchun 130024 China

**Keywords:** carbon dots, interfacial energy storage, lithium ion batteries, manganese‐vanadium‐oxo clusters, monodisperse

## Abstract

Although possessing well‐defined nanostructures and excellent multi‐electron redox properties, polyoxometalate clusters have poor intrinsic electrical conductivity and are prone to aggregation due to large surface energy, which makes them difficult to be fully utilized when applying as electrode materials for lithium‐ion batteries. In this paper, monodisperse K_7_MnV_13_O_38_ (MnV_13_) clusters are achieved by rationally utilizing nano‐sized high conductive carbon dots (CDs) as stabilizers. Benefiting from the fully exposed redox sites of MnV_13_ clusters (high utilization rate) and sufficient interfaces with carbon dots (extra interfacial energy storage), the optimized MnV_13_/10CDs anode delivers a high discharge capacity up to 1348 mAh g^−1^ at a current density of 0.1 A g^−1^ and exhibits superb rate/cycling capabilities. Density functional theory (DFT) calculations verify that ionic archway channels are formed between MnV_13_ and CDs, eliminating the bandgap and greatly improving the electron/ion conductivity of MnV_13_ and CDs. This paper paves a brand‐new way for synthesis of monodisperse clusters and maximization of extra interfacial energy storage.

## Graphical abstract

The article achieves monodispersion of metal‐oxygen clusters by the freeze‐drying method using nano‐sized carbon dots (CDs). It solves the problems of intrinsic low conductivity, easy aggregation, and poor stability of metal‐oxygen clusters. The high utilization rate and additional interfacial energy storage significantly improve the multiplicity and cycle stability of lithium‐ion batteries while endowing them with high‐density energy storage.

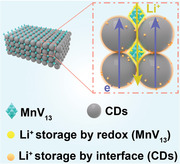

## 1 Introduction

**Figure 1:**
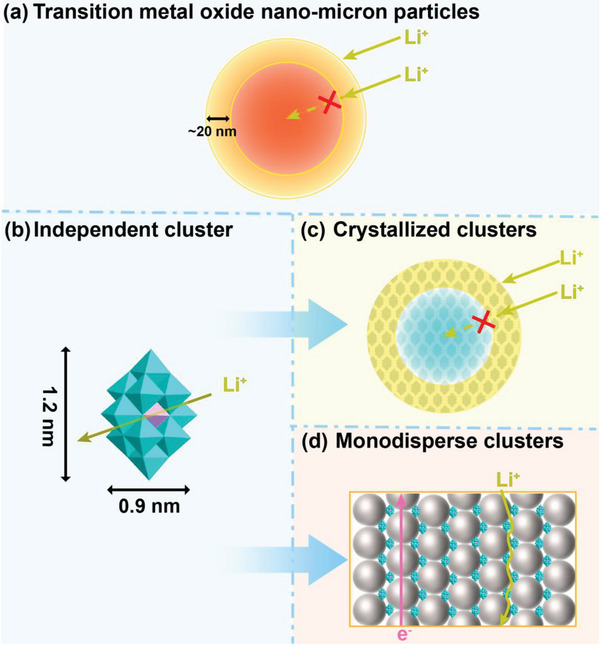
Schematic illustration of the transport of lithium ions in the species of a) Transition metal oxide nanoparticles, b) Independent cluster, c) Crystallized clusters, and d) Monodisperse clusters.

Lithium‐ion batteries (LIBs) are characterized by high energy density, long lifespan, environmental friendliness, and widely used in portable electronic products and energy conversion systems for electric vehicles.^[^ ^1^ ^]^ So far, among various anode materials for LIBs, transition metal oxides (TMOs)^[^ ^2^ ^]^ have attracted much attention due to their high theoretical capacity relative to the carbon‐based counterparts,^[^ ^3^ ^]^ such as vanadium oxides (VO*_x_*)^[^ ^4^ ^]^ and manganese oxides (MnO*_x_*),^[^ ^5^ ^]^ etc. However, the universally utilized TMOs are generally in scale of several micrometers or hundreds of nanometers, while the effective transport distance of lithium ions in LIBs is generally less than 20 nanometers.^[^ ^6^ ^]^ This indicates that most of the inner part of TMOs is not involved in the reaction, which not only wastes resources but also cripples the overall capacity of the LIBs^[^ ^7^ ^]^ (**Figure** **1**a). Although TMOs can be downsized to less than 20 nanometers by strategies of using surfactants or adopting active group protection, this undoubtedly increases the cost and the complexity of the process. In addition, the added surfactants can hinder the efficient transportation of lithium ions. Therefore, employing materials with small intrinsic sizes could be a major breakthrough in solving the above problems.

Clusters, as important mesoscopic species bridging the gap between macro/nanomaterials and microscopic atoms, inherit the advantage of high theoretical specific capacity of TMOs and show promising prospects in the field of energy storage and conversion in virtue of their intrinsic sub‐nanometer sizes^[^ ^8^ ^]^ (Figure 1b). However, in practical applications, clusters tend to agglomerate into crystalline states due to extremely high surface energy, resulting in dramatically decreased accessible surface area and active sites^[^ ^9^ ^]^ (Figure 1c). To circumvent these dilemmas, a judicious strategy is to implement stable monodispersion of clusters. Carbon dots (CDs) are an emerging nanoscale carbon‐based material with abundant oxygen‐containing functional groups on its surface.^[^ ^10^ ^]^ They can stabilize various clusters by displacing partial oxygen atoms on the clusters or forming supramolecular structures to monodisperse the clusters so that sufficient active sites can be well exposed for lithium storage. In addition, CDs have excellent electron transfer ability, which can improve the electrical conductivity of the material.^[^ ^11^ ^]^ The voids formed by the dense stacking of CDs can trigger additional interfacial effects and promote the storage of lithium ions. In view of these advantages, dispersed clusters using CDs are expected to significantly improve the performance of LIBs.

According to the above analysis, a readily accessible paradigm for monodispersing POM clusters is proposed in this study. K_7_MnV_13_O_38_ clusters (denoted as MnV_13_) are adopted as a representative model system and achieved their good monodispersity with the help of nano‐sized highly conductive CDs using a facile freeze‐drying process. (Figure 1d) By rationally tuning the concentration of MnV_13_ and CDs, the MnV_13_ clusters are well monodispersing on CDs and thus can fully expose their redox active sites to the environment and possess greatly expanded interfacial area for extra energy storage relatives to their bulk counterparts, which can dramatically boost their electrochemical performances when applying as anode materials for LIBs. When the mass ratio of MnV_13_ to CDs reaches 1:10, that is, MnV_13_/10CDs, a discharge capacity of 1348 mAh g^−1^ is achieved at a current density of 0.1 A g^−1^, with excellent cycling stability and Couloumbic efficiency. Moreover, density functional theory (DFT) calculations verify that ionic archway channels are formed between MnV_13_ and CDs, eliminating the bandgap and greatly improving the electron/ion conductivity of MnV_13_ and CDs. This study provides a facile, quick, and effective approach to achieving monodispersed POM clusters as well as triggering desirable and considerable interfacial energy storage.

## 2 Results and Discussion

### 2.1 Synthesis and Structural Characterization of Monodisperse K_7_MnV_13_O_38_

**Figure 2:**
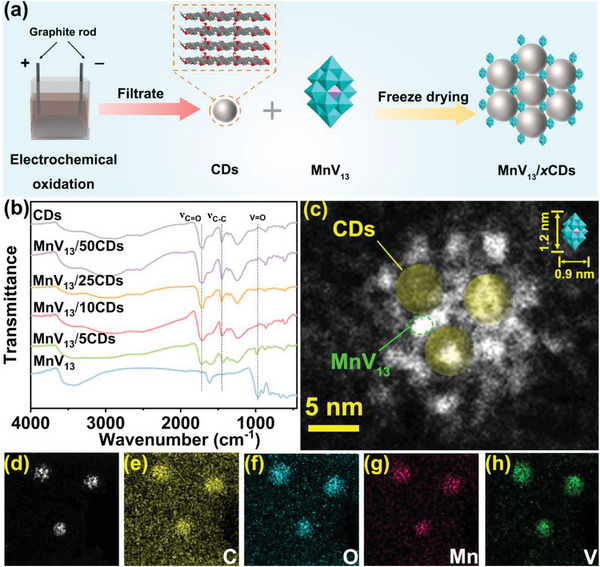
a) Synthetic diagram of MnV_13_/*x*CDs specimens. b) FT‐IR spectra of MnV_13_/*x*CDs specimens and bare MnV_13_ and CDs. c) HAADF‐STEM image of MnV_13_/10CDs. d) HRTEM image of MnV_13_/10CDs. e–h) Elemental mapping of C, O, Mn, and V elements.

The synthetic route of monodisperse MnV_13_ clusters in CDs is shown in **Figure** **2**a. CDs^[^ ^10c^ ^]^ and MnV_13_^[^ ^12^ ^]^ are synthesized according to the literatures. Detailed synthesis methods can be referred in the Supporting Information and the relevant characterizations are shown in Figure S1−S3 (Supporting Information). The CDs display well‐disperse morphology while the bare MnV_13_ shows in the bulk form. After homogeneously mixing the CDs solution with the MnV_13_ solution and suffering the subsequent freeze‐drying process, the MnV_13_/*x*CDs specimens, *x* is the mass ratio of CDs to MnV_13_ clusters, can be obtained. Benefiting from the efficacy of CDs serving as stabilizers to greatly suppress the undesired aggregation of MnV_13_, MnV_13_ clusters can well monodisperse in CDs. However, the content of CDs needs to be precisely regulated. Figure 2b compares the Fourier transform infrared spectroscopy (FT‐IR) of the MnV_13_/*x*CDs specimens as well as bare MnV_13_ and CDs. With the increasing proportion of CDs, the characteristic vibrational peak of MnV_13_ at 990 cm^−1^ gradually weakens while ones of CDs at 1700 and 1450 cm^−1^ strengthen. When the mass ratio of MnV_13_ to CDs reaches 1:10, the characteristic vibrational peaks of MnV_13_ are almost invisible. Figure S4 (Supporting Information) compares the X‐ray diffraction (XRD) patterns of the MnV_13_/*x*CDs specimens as well as bare MnV_13_ and CDs. It can be seen that the MnV_13_ possesses sharp characteristic peaks, demonstrating its crystalline nature. While after mixing with CDs, these sharp peaks gradually disappear and only a bun‐like peak located at ≈25° can be seen, indicating that MnV_13_ clusters are well dispersed in the CDs. High‐angle annular dark‐field scanning transmission electron microscopy (HAADF‐STEM) is further conducted to reveal the actual nanostructure of the MnV_13_/10CDs specimen. As shown in Figure 2c and Figure S5 (Supporting Information), MnV_13_ and CDs are connected by hydrogen bonds to form a spherical structure. The nanoparticles have a mean corresponding size of ≈1.5 nm, which match well to the MnV_13_ clusters with a length of 1.2 nm and a width of 0.9 nm, verifying the monodispersion of MnV_13_ clusters in CDs. Furthermore, elemental mapping reveals the co‐existence and uniform distribution of C, O, Mn, and V elements, indicating the well‐dispersing of MnV_13_ clusters, as shown in Figure 2d–h.

### 2.2 Electrochemical Performance

**Figure 3:**
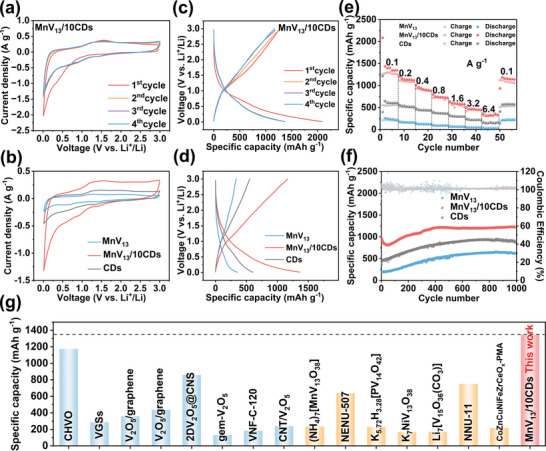
a) The first four C–V curves of MnV_13_/10CDs at 0.2 mV s^−1^ within 0.01−3.0 V. b) Comparison of C–V curves of MnV_13_/10CDs, bare MnV_13_ and CDs at 0.2 mV s^−1^. c) The first four GCD curves of MnV_13_/10CDs at a current density of 0.1 A g^−1^. d) Comparison of GCD curves of MnV_13_/10CDs, bare MnV_13_ and CDs at 0.1 A g^−1^. e) Rate performance of MnV_13_/10CDs, bare MnV_13_ and CDs. f) Cycling performance of MnV_13_/10CDs, bare MnV_13,_ and CDs at 1 C for 1000 cycles. g) Comparison of the specific capacity of MnV_13_/10CDs in this study with that of vanadium‐based electrode materials and cluster materials recently reported for LIB applications.

To evaluate the lithium storage performance of MnV_13_/*x*CDs specimens, a series of coin‐type LIBs are assembled. The first four cyclic voltammetry curves (CV) of the MnV_13_/10CDs electrode at a scan rate of 0.2 mV s^−1^ are shown in **Figure** **3**a. It can be clearly seen that the first CV cycle significantly differs from the subsequent cycles, which is due to the formation of a solid‐electrolyte interphase (SEI) film on the electrode surface during the Li^+^ insertion process. The curves show two pairs of reversible redox peaks at 0.92/1.56 V and 1.47/2.3 V starting from the second cycle. Figure 3b and Figure S6 (Supporting Information) compare the C–V curves of the MnV_13_/*x*CDs specimens with bare MnV_13_ and CDs. Among them, MnV_13_/10CDs exhibit the largest current density and area of integration, suggesting that it has the highest capacity. Figure 3c shows the galvanostatic charge‐discharge (GCD) curves of the MnV_13_/10CDs over the first four cycles at a current density of 0.1 A g^−1^. The specific capacity of MnV_13_/10CDs is 2097 and 1348 mAh g^−1^ during the first discharge and charge, respectively, with a Coulombic efficiency of 64.3%. The reason for the low initial coulombic efficiency is the formation of SEI film on the electrode surface. However, in subsequent cycles, the Coulombic efficiency quickly approaches ≈100%, indicating the excellent reversibility of the material. Additionally, the discharging curve exhibits two plateaus at 0.9–1.5 V, while the charging branch displays another two plateaus at 1.5–2.3 V, which align well with the redox peaks in the C–V curves. Figure 3d and Figure S7 (Supporting Information) compare the fourth cycle of charge–discharge curves at a current density of 0.1 A g^−1^ for MnV_13_/*x*CDs as well as bare MnV_13_ and CDs electrodes. The reversible discharge specific capacity of MnV_13_/10CDs, bare MnV_13_, and CDs is 1348, 253, and 604 mAh g^−1^, respectively. The achievement of the highest capacity of MnV_13_/10CDs is attributed to the even dispersion of MnV_13_ clusters on CDs and the exposure of more active sites on the surface. However, insufficient CDs fail to inhibit the aggregation of MnV_13_ while excess CDs will sacrifice the overall capacity of the electrode system due to the low intrinsic theoretical specific capacity of CDs. Figure 3e and Figure S8 (Supporting Information) investigate the rate performance of MnV_13_/*x*CDs as well as bare MnV_13_ and CDs electrodes. Due to the more active site exposure and interfacial energy storage effects, MnV_13_/10CDs can store more Li^+^ ions and trigger their fast transport. MnV_13_/10CDs electrode delivers a specific capacity of 1348, 1119, 885, 724, 579, 449, and 337 mAh g^−1^ at a current density of 0.1, 0.2, 0.4, 0.8, 1.6, 3.2, and 6.4 A g^−1^, respectively, which are significantly higher than those of bare MnV_13_, CDs and other MnV_13_/*x*CDs counterparts. When the current density is restored to the initial 0.1 A g^−1^, the capacity of MnV_13_/10CDs recovers to 83.6% of the original value. This suggests that MnV_13_ clusters dispersed by CDs possess outstanding structural stability and superb rate capability.

To further evaluate the long‐term stability of the electrodes, 1000 charge/discharge cycles at a current density of 1 C (0.8 A g^−1^) are performed. As shown in Figure 3f and Figure S9 (Supporting Information), the capacity of MnV_13_/10CDs shows a slightly drop in the initial 50 cycles due to the replacing process of internal K^+^ ions by surrounding Li^+^ ions, and then gradually increases in the subsequent 350 cycles for activation process and finally reaches a stable state. After 1000 cycles, the capacity of MnV_13_/10CDs achieves ≈1250 mAh g^−1^. It is worth noting that during the 1000 cycles, although both the capacity of bare MnV_13_ and CDs also display a gradually increasing trend, the underlying mechanism is quite different. The gradually increasing trend of bare CDs capacity mainly attributes to the enhancement of wettability between the CDs, while that of bare MnV_13_ is mainly due to the capacity contribution of electrolyte decomposition on the newly formed surface by the continuous pulverization of MnV_13_ bulk. Figure 3g compares the capacity of MnV_13_/10CDs in this work with other representative counterparts based on V_2_O_5_ nanocomposites and polyvanadates reported in recent years. Our MnV_13_/10CDs exhibit the highest capacity due to the synergistic effect of monodispersion of MnV_13_ clusters and the inducing interfacial energy storage.

### 2.3 Reaction/Diffusion Kinetic Mechanism

**Figure 4:**
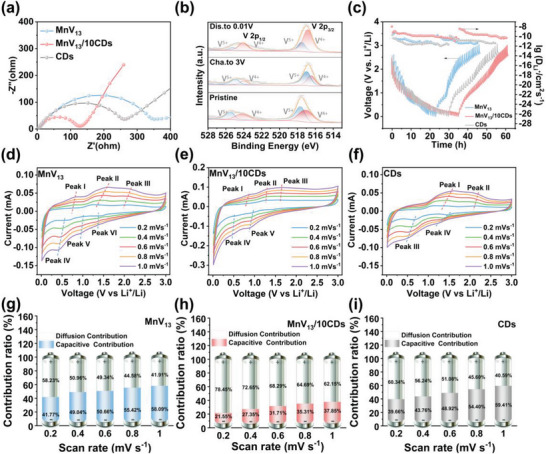
a) EIS measurements of MnV_13_/10CDs, bare MnV_13_ and CDs electrodes. b) XPS spectra of V 2p for MnV13 electrodes in the pristine state, discharged to 0.01 V and charged to 3 V. c) GITT plots and the calculated *D*_Li_^+^ values of the MnV_13_/10CDs, bare MnV_13_ and CDs electrode during discharging and charging. C–V curves of d) MnV_13_, e) MnV_13_/10CDs, and f) CDs at different scan rates. Diffusion contribution and Capacitive contribution of g) MnV_13_, e) MnV_13_/10CDs, and f) CDs at various scan rates.

In order to reveal the diffusion kinetics of lithium ions within the hybrid electrodes as well as the charge transfer at the electrode/electrolyte interface, we performed electrochemical impedance spectroscopy (EIS) studies on MnV_13_/10CDs, bare MnV_13_, and CDs electrodes in the frequency range of 100 kHz to 10 MHz. The Nyquist plot consists of three parts. Among them, the intercept on the X‐axis represents the intrinsic resistance (*R*_I_) of the electrolyte and electrode, and the diameter of the semicircle represents the charge transfer resistance (*R*_CT_) when electrons and lithium ions pass through the electrode/electrolyte interface. The slope of the line represents the Warburg resistance (*Z*_w_) in the electrode related to lithium‐ion diffusion. The MnV_13_/10CDs electrode shows the smallest *R*_I_, *R*_CT,_ and steepest curves before cycling, indicating the least resistance to lithium‐ion diffusion (**Figure** **4**a). As shown in Figure S10 (Supporting Information), the smallest change of *R*_CT_ of MnV_13_/10CDs before and after 1000 cycles verifies its best structural stability. The valence state changes of MnV_13_ during charging and discharging are analyzed by X‐ray photoelectron spectroscopy (XPS). As shown in Figure 4b, the pristine MnV_13_ has peaks at 525.5 and 524.1 eV (V 2*p*_1/2_) as well as 517.7 and 517.0 eV in the V 2*p*_3/2_ region, respectively, which are assigned to V^5+^ and V^4+^. During discharge, the intensity of V^5+^ decreases while the intensity of V^4+^ slightly increases as Li^+^ inserts. When the electrode is charged to 3 V, V^4+^ is oxidized and thus the intensity of V^5+^ increases, approaching the intensity of the initial state, indicating the reversible redox process of charging/discharging. These phenomena confirm the structural stability of the MnV_13_/10CDs during Li^+^ insertion and extraction.

The lithium‐ion diffusion coefficients during charging and discharging were evaluated using the galvanostatic intermittent titration technique (GITT). The GITT curves and corresponding Li^+^ ion diffusion coefficients of MnV_13_/*x*CDs specimens as well as bare MnV_13_ and CDs are shown in Figure 4c and Figure S11 (Supporting Information), respectively. According to Fick's second law, the diffusion coefficient of Li^+^ ions (*D*_Li_^+^) in the electrode can be calculated:

(1) $$D_{Li^{+}}=\frac{4}{\pi \tau }\;{\left(\frac{m_{B}V_{M}}{M_{B}S}\right)}^{2}{\left(\frac{\Delta E_{s}}{\Delta E_{\tau }}\right)}^{2}$$

where *τ* is the excitation current time (s); *m*_B_, *V*_M_, and *M*_B_ are the mass (g), molar volume (cm^3^ mol^−1^), and molecular weight (g mol^−1^) of the MnV_13_/*x*CDs, respectively; *S* is the electrode area (cm^2^); Δ*E*_S_ is steady‐state voltage change, Δ*E*_τ_ is the transient voltage variable. A pulse current of 0.1 A g^−1^ is used with a charge/discharge time of 10 min and 60 min of standing time. From the figure, it can be seen that the ion diffusion coefficient of MnV_13_/10CDs is the highest in the range of 10^−8.5^–10^−10.9^, indicating that MnV_13_/10CDs has both excellent redox activity and interface structure, providing more metallic active sites for the Li^+^ storage and improving the diffusion kinetics of Li^+^ ions. To further reveal their electrochemical reaction kinetics, we comprehensively evaluated the capacitive charge storage and diffusion control processes at MnV_13_/10CDs, bare MnV_13,_ and CDs hybrid electrodes by measuring the CVs at different scan rates.

The results are shown in Figure 4d–f and Figure S12 (Supporting Information). When the scan rate is increased from 0.2 to 1.0 mV s^−1^, the C–V curves exhibit similar shapes with increasing peak intensity. In the C–V curves, the peak current follows a rule that increases with the scan rate

(2) $$i=av^{b}$$

where *i* is the peak current, *v* is the scan rate, as well as *a* and *b* are variable parameters. In our work, as shown in Figure S13 (Supporting Information), the *b* values of MnV_13_/*x*CDs specimens are between 0.5 and 1, indicating that both diffusion control and capacitance control are present in the Li^+^ insertion/extraction process. In addition, the capacitance contribution to the total capacity can be calculated by the following equation:

(3) $$i\;\left(V\right)=k_{1}v+k_{2}v^{1/2}$$

where *k*_1_ *v* and *k*_2_ *v* ^1/2^ are the assigned values for the capacitive and diffusion control processes, respectively. The calculated capacitive and diffusive contributions at different scan rates are shown in Figure 4g–i and Figure S14 (Supporting Information). At scan rates of 0.2, 0.4, 0.6, 0.8, and 1.0 mV s^−1^, the capacitance contributions of the MnV_13_/10CDs electrode are 21.55%, 27.35%, 31.71%, 35.31%, and 37.85%, respectively, which are lower than those of the corresponding bare MnV_13_ and CDs. This indicates that introducing CDs to monodisperse MnV_13_ can simultaneously boost the capacitive charge storage (more interfacial surface) and redox reactions (short ion diffusion distance and high utilization rate). Based on the calculation from CV, the specific capacity of MnV_13_/10CDs at 0.2 mV s^−1^ is 882 mAh g^−1^, which is much higher than the theoretical capacity of 465 mAh g^−1^. This indicates that the interfacial effect in the hybrid electrode contributes a significant portion of the capacity. At 1.0 mV s^−1^, the redox contribution of MnV_13_/10CDs is 319 mAh g^−1^, which is quite close to the theoretical contribution value of 347 mAh g^−1^, suggesting that the MnV_13_ cluster can be also taken full advantage even under a fast charging and discharging rate.

**Figure 5:**
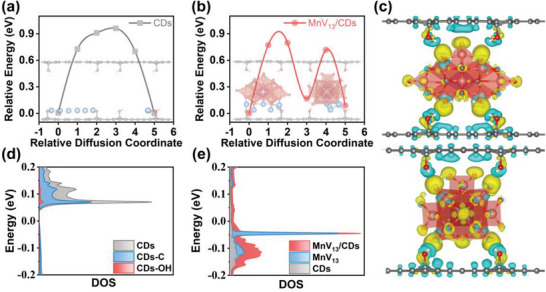
Simulated migration energy barrier of Li^+^ ions in a) bare CDs and b) MnV_13_/CDs; c) Differential charge density of MnV_13_/CDs system (gray, red, pink and white atoms represent C, O, V, Mn, and H atoms, and blue and yellow outlines represent charge loss and charge accumulation); The total DOS and pDOS of d) bare CDs and e) MnV_13_/CDs near Fermi level.

First‐principles density functional theory (DFT) simulations are performed to in‐depth uncover the interaction between monodispersed MnV_13_ and CDs by using MnV_13_/CDs and CDs as typical models. The ease of ion migration on the surface of the bulk material is a key factor in determining the electrochemical performance of the electrode materials, as shown in **Figure** **5**a,b. When Li^+^ ions diffuse along the surface of the MnV_13_/CDs, the migration barrier is 0.78 eV, much lower than that of bare CDs of 0.92 eV. This is due to the electron transfer that occurs between MnV_13_ and CDs through hydrogen bonds forms charged ion “archway” channels that facilitate fast ion transport (Figure 5c). In addition, the electron transfer resistance within the bulk material is another key factor in the electrochemical performance of the electrode material. When MnV_13_ is adsorbed on the surface of CDs, it can enhance the non‐uniform distribution of charges in CDs, increase the density of states at the Fermi level, and eliminate the bandgap in CDs, making MnV_13_/CDs exhibit a conductive form (Figure 5d,e). Therefore, the interaction between MnV_13_ and CDs is not simple adsorption, but occurs through electron transfer via hydrogen bonds, forming ion “archway” channels and eliminating the bandgap, greatly improving the electron/ion conductivity of MnV_13_ and CDs. This is well consistent with the experimental results.

## 3 Conclusion

In summary, monodisperse MnV_13_ clusters are achieved by introducing nano‐sized CDs with highly electronic conductivity in a facile freeze‐drying method. Structural characterizations and DFT calculations verify that the interaction between MnV_13_ and CDs is not simple adsorption but instead involves an electron transfer through hydrogen bonds, forming ion “archway” channels and eliminating the bandgap, which greatly improves the electronic/ionic conductivity of MnV_13_ and CDs. Benefiting from these merits, the LIB based on the MnV_13_/10CDs hybrid electrode exhibits a reversible specific capacity of up to 1348 mAh g^−1^ at a current density of 0.1 A g^−1^ and superior cycling performance. This research provides a novel approach for the realization of monodispersed POM clusters and maximized interfacial energy storage.

## Conflict of Interest

The authors declare no conflict of interest.

## Supporting information

Supporting Information

## Data Availability

The data that support the findings of this study are available from the corresponding author upon reasonable request.
