# Supplementary material for: Monodisperse Manganese‐Vanadium‐Oxo Clusters with Extraordinary Lithium Storage
Source: Adv Sci (Weinh). 2024 Jun 3;11(29):2402616. doi: 10.1002/advs.202402616 (PMC11304304; doi:10.1002/advs.202402616)
Supplement: Supplementary file 1 — Supporting Information [file ADVS-11-2402616-s001.docx]

**Monodisperse Manganese-Vanadium-Oxo Clusters with Extraordinary Lithium Storage**

*Wensi Tang*^†^*, Tianyu Qiu*^†^*,* *Zhiyuan Hu, Yingqi Li*, Ruiqi Yao, Yonghui Wang*, Xingyou Lang, Huaqiao Tan**, *Yangguang Li, Qing Jiang*

W. S. Tang, T. Y. Qiu, Z. Y. Hu, Y. Q. Li, R. Q. Yao, Y. H. Wang, Y. G. Li, H. Q. Tan

Key Laboratory of Polyoxometalate and Reticular Material Chemistry of Ministry of Education, Faculty of Chemistry, Northeast Normal University, Changchun, Jilin 130024, China.

X. Y. Lang, Q. Jiang

Key Laboratory of Automobile Materials (Jilin University), Ministry of Education and School of Materials Science and Engineering, Jilin University, Changchun, 130024, China.

E-mail: [liyq164@nenu.edu.cn](mailto:liyq164@nenu.edu.cn) (Y. Q. Li), [wangyh319@nenu.edu.cn](mailto:%20wangyh319@nenu.edu.cn) (Y. H. Wang) and [tanhq870@nenu.edu.cn](mailto:tanhq870@nenu.edu.cn) (H. Q. Tan)

† These authors contributed equally to this work.

⁎ Corresponding author

**Materials and Methods**

Synthesis of CDs

In a glass container with the number 40 cm *40 cm *40 cm, 48 liters of deionized water is poured in, and 64 spectrographic grade carbon rods are alternately arranged with positive and negative poles. A voltage of 30V is applied to the positive and negative poles until the current reaches 0.8A, and the resulting brown solution is the carbon dot solution^[1]^.

Synthesis of MnV_13_

The POM was synthesized according to a reported literature^[2]^. 17.95 g (0.130 mol) potassium metavanadate was dissolved in 500 mL of hot water and 10 mL of 0.5 M sulfuric acid was added to the resulting light yellow solution. Then 0.01 mol of manganese(II) sulfate and 5.40 g (0.020 mol) of potassium peroxydisulfate were added sequentially with continued stirring and heating. When the mixture was evaporated to a volume of about 150 mL, the opaque brown-black mixture was heated almost to boiling under vigorous stirring and immediately filtered. To this black filtrate, 20 ml of 1 M potassium acetate was added and the filtrate was heated again almost to boiling in order to redissolve the precipitate that started to form when the solution was cooled. After the solution cools to room temperature, the red-orange crystals MnV_13_ were obtained. The relevant characterization is shown in Figure S2.

Synthesis of MnV_13_/*x*CDs

MnV_13_ were dissolved in CDs solution and stirred thoroughly. The mixed solution was then freeze-dried. The final products were recorded as MnV_13_/*x*CDs (*x* means the mass ratio of CDs to MnV_13_).

**Materials Characterization**

The interrelated energy dispersive X-ray detector (EDAX) spectra and Scanning Electron Microscopy (SEM) of graphite felt electrode was achieved by using a SU8000 ESEM FEG microscope. The powder X-ray diffraction (PXRD) measurements were carried out on a Rigaku D/max-IIB X-ray diffractometer with Cu-Kα radiation (λ=1.5418 Å). The structural information of the specimens was analyzed by Fourier Transform Infrared Spectrometer (FT-IR) (BRUKE Vertex 70, 0.4 cm^-1^, 400-4000 cm^-1^). X-ray photoelectron spectrum (XPS) analyses were performed on an ESCALABMKII spectrometer with an Al-Kα (1486.6 eV) achromatic X-ray source. Transmission electron microscopy (TEM) and HRTEM images were measured on a JEOL-2100F microscope operated at 200 kV.

**Electrochemical Measurements**

In order to obtain electrode for electrochemical test, slurry was composed of 80 wt% active materials, 10 wt% poly-vinylidene fluoride (PVDF), and 10 wt% acetylene black in (N-Methylpyrrolidone) NMP. After the slurry was well mixed, it was coated on copper foil and dried in vacuum at 80 °C for 10 h. Coin-type (CR2025) cells were assembled in an Ar-filled glove box (H_2_O < 0.1 ppm, O_2_ < 0.1 ppm) with lithium metal foil as the counter/reference electrode and Canrd2500 as the separator. The electrolyte was 1 M lithium hexafluorophosphate (LiPF6) dissolved in EC: EMC: DMC (v/v = 1:1:1). 10 μL of electrolyte was added into each cell. The assembled cell was kept in Humidity Chamber for 24 h prior to the test. The coin cells were tested in galvanostatic mode at various current densities within a voltage range of 0.01 to 3.0 V using LANDdt multichannel battery test system. Cyclic voltammogram (CV) and electrochemical impedance spectroscopy (EIS) measurements were performed on Squidstat Plus electrochemical workstation (Admiral Instruments) at a scan rate of 0.2 mV/s. EIS test was conducted in frequency range of 100 kHz to 10 mHz. All specific capacities were calculated based on the mass of MnV13/*x*CDs.

**Calculation detail**

First-principles calculations on the basis of density functional theory (DFT) were performed by using projector augmented wave (PAW) method as implemented in Vienna ab initio simulation package (VASP) with a plane-wave basis set. The functional of Perdew, Burke, and Ernzerhof (PBE)^[3]^ was used to describe the exchange-correlation energy. The DFT calculation was used to optimize the structure and study the differential charge density. A 450 *e*V cutoff was employed for all the computations. A Monkhorst-Pack k-point mesh of 1×1×1 was used for MnV_13_+CDs and CDs unit cell. The MnV_13_ cluster structural models are analyzed by single crystal X-ray and are all fully optimized until the forces are less than 10^-2^ *e*V/A and the energy converges to 10^-5^ *e*V between the two successive self-consistent steps. Evaluation of Li-ion migration behavior in different regions of the model using climbing-image NEB method^[4]^.

**Supplementary Figures**


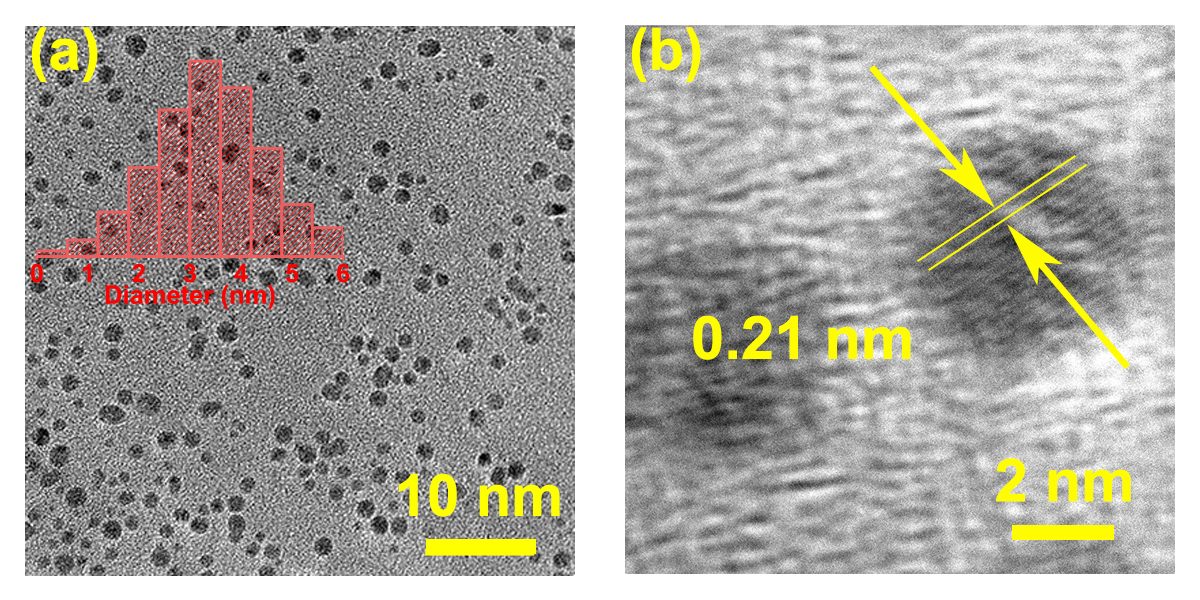


**Figure S1**. TEM and HRTEM images of CDs.

As shown in **Figure S1a**, the synthesized carbon dots have a uniform size with a diameter of about 2-5 nm. The lattice stripes of the carbon dots are approximately 0.21 nm, which are consistent with the literatures.

.
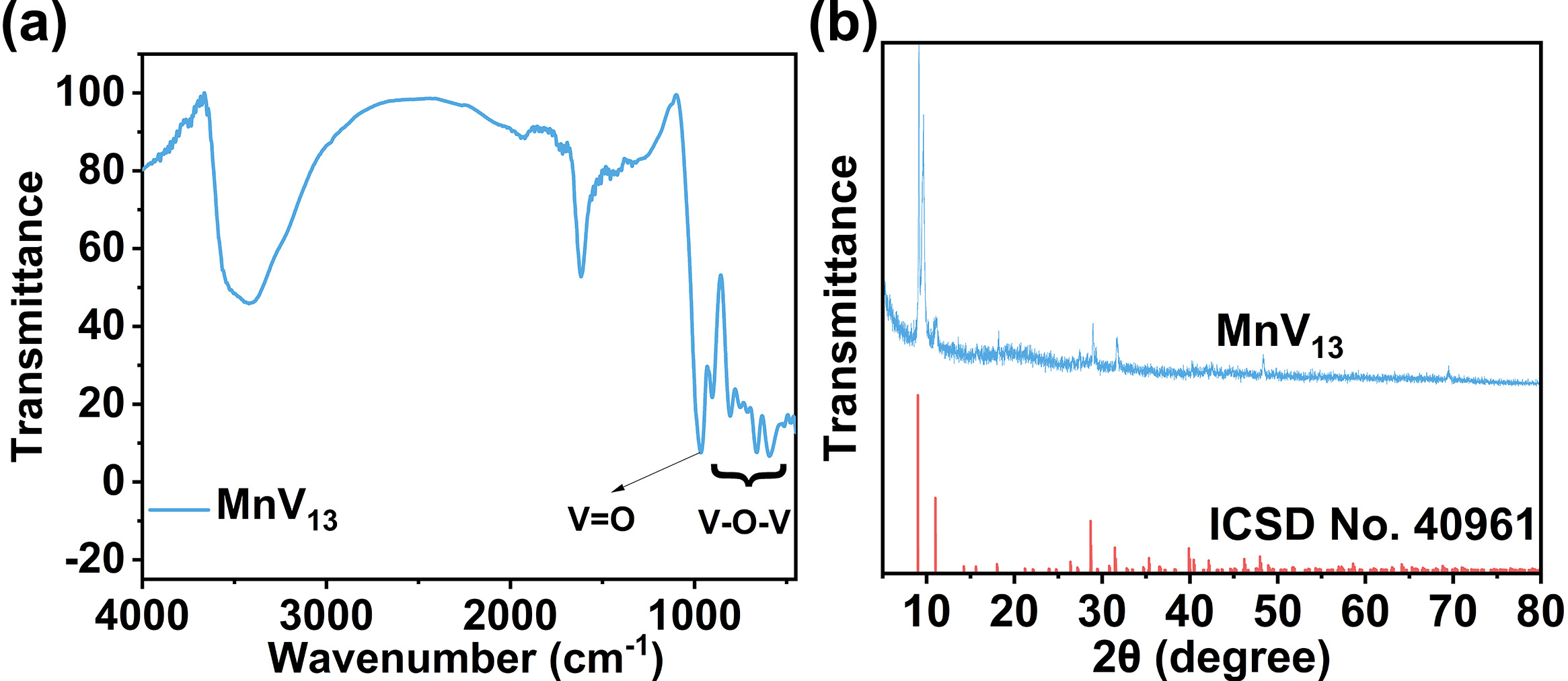


**Figure S2**. (a) FT-IR spectrum of MnV_13_. (b) XRD pattern of MnV_13._

**Figure S2a** shows the FT-IR spectrum of MnV_13_, with a V=O peak at 959 cm^−1^ and vibration peaks of V-O-V at 500-800 cm^−1^. **Figure S2b** shows the XRD pattern of MnV_13_, which is basically consistent with ICSD No. 40961, indicating the successful synthesis of MnV_13_.


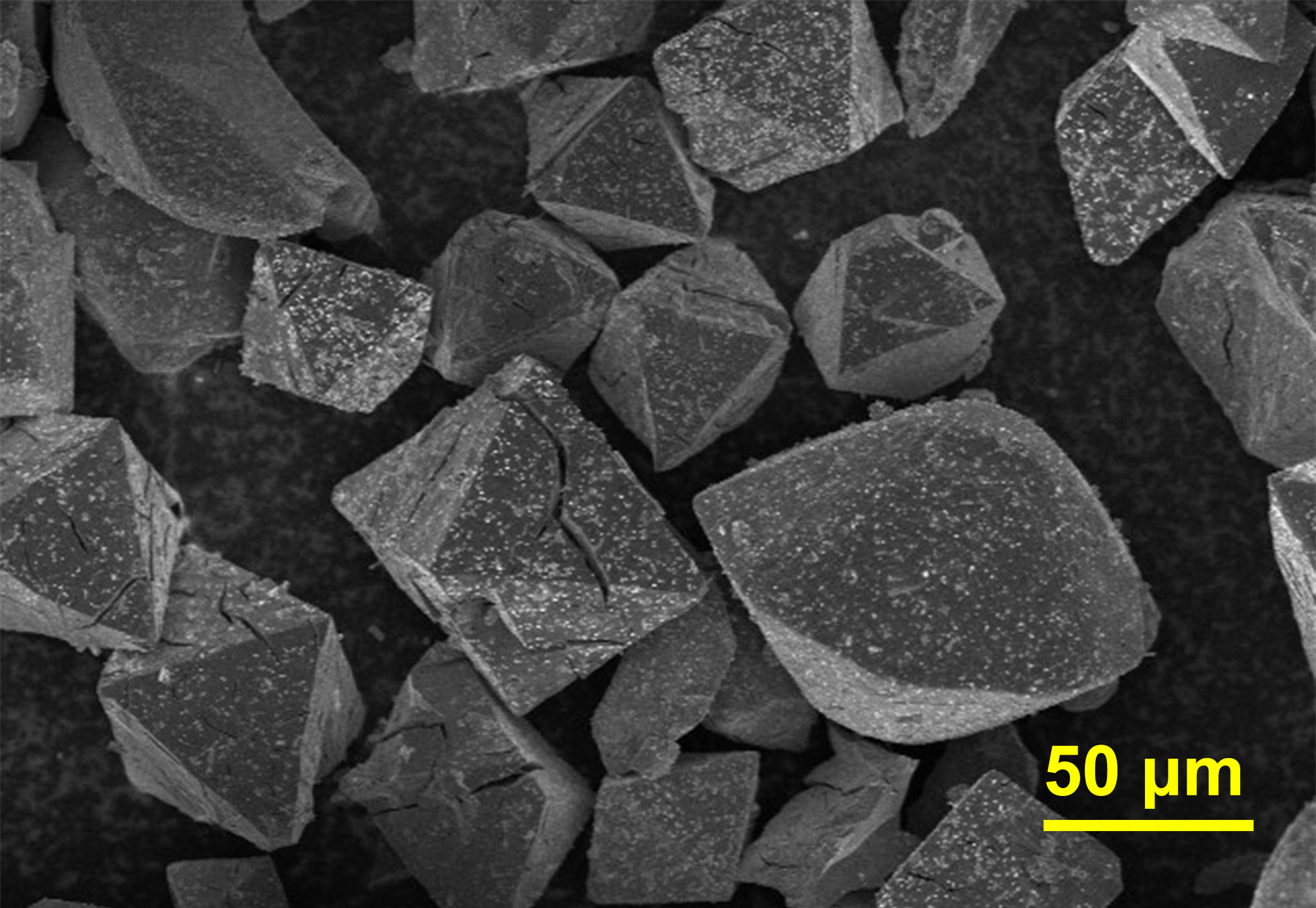


**Figure S3.** SEM image of MnV_13_.

The bare MnV_13_ shows in the bulk form.


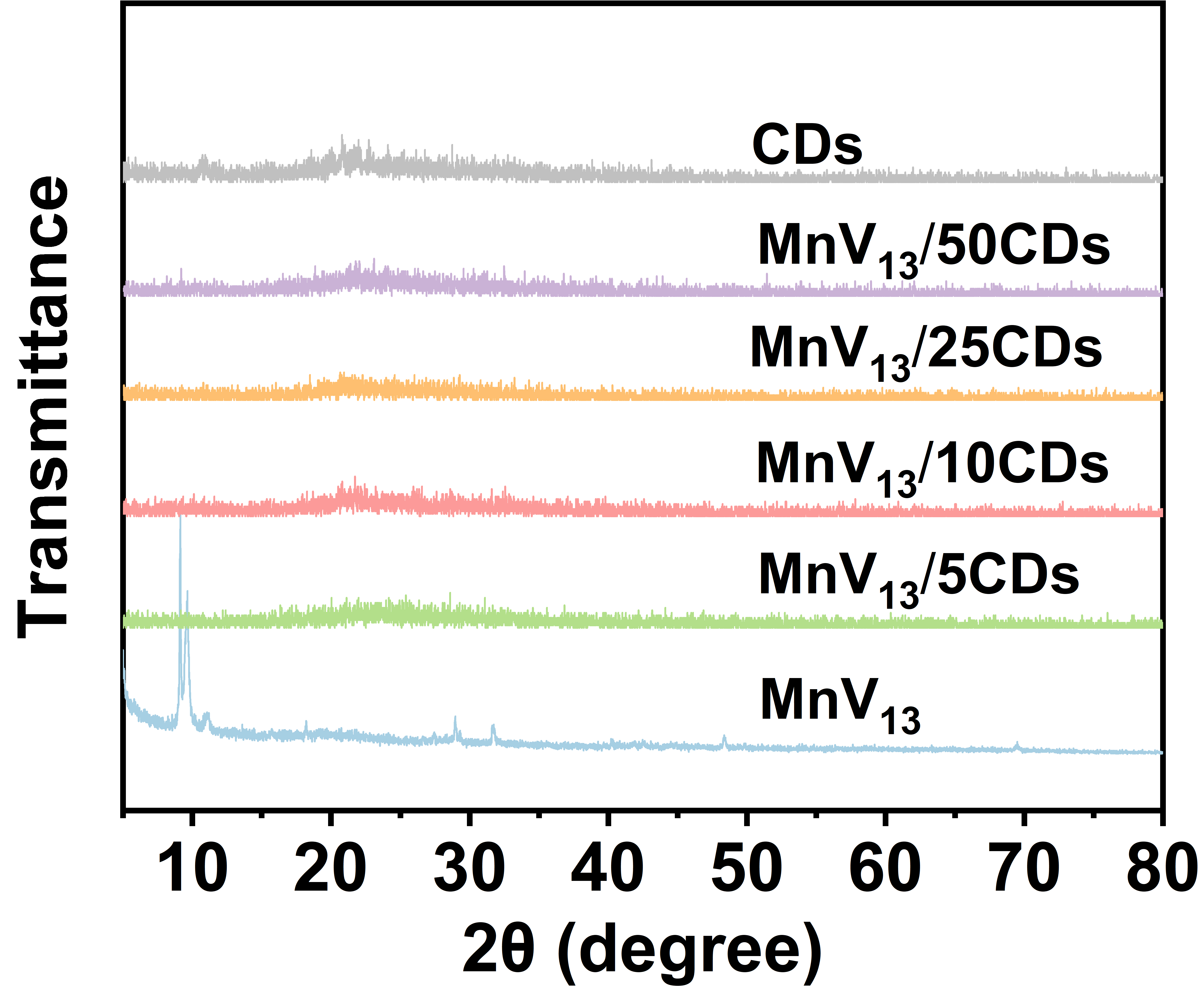


**Figure S4**. XRD pattern of MnV_13_/*x*CDs.


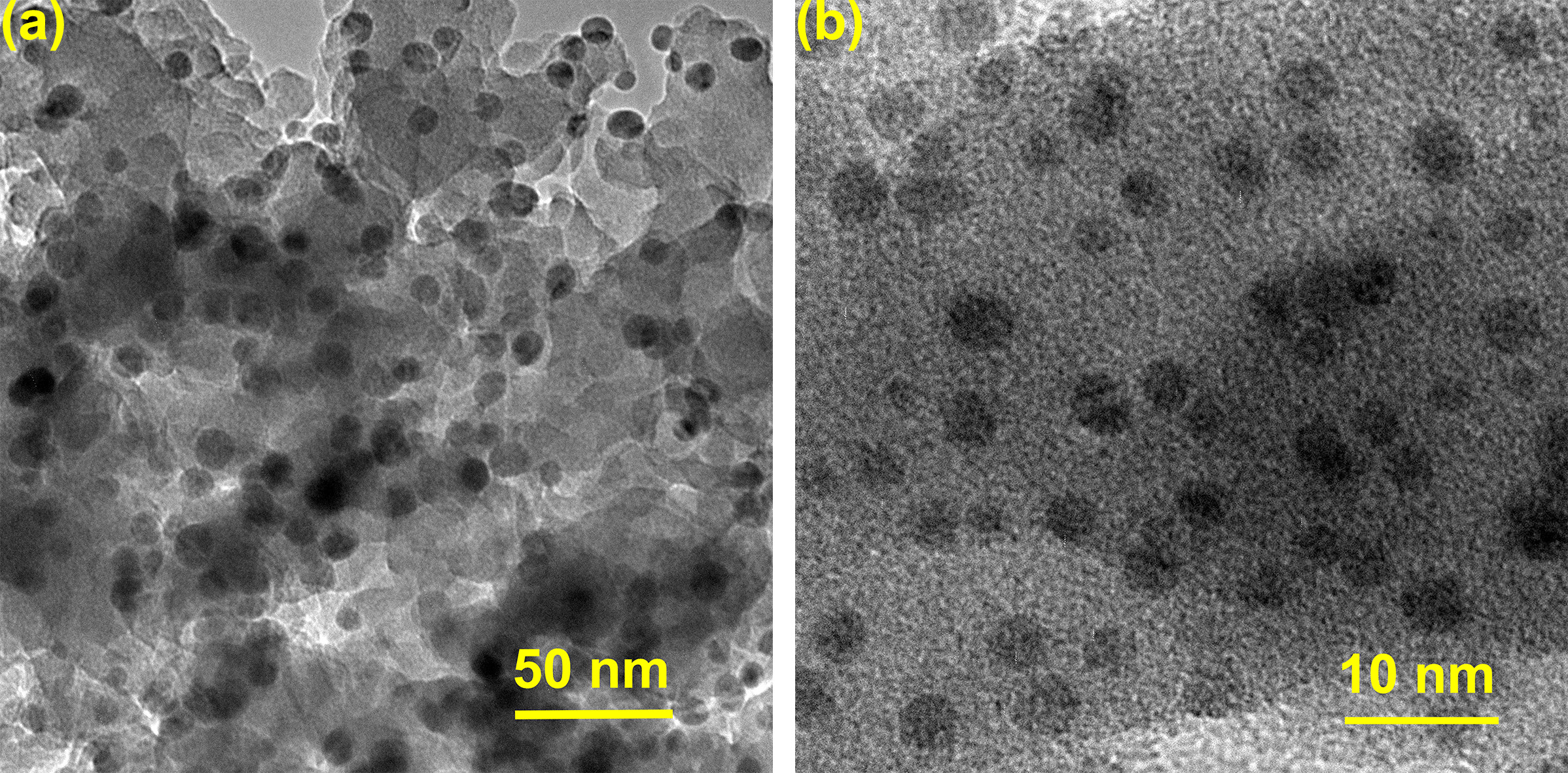


**Figure S5**. TEM image of MnV_13_/10CDs.


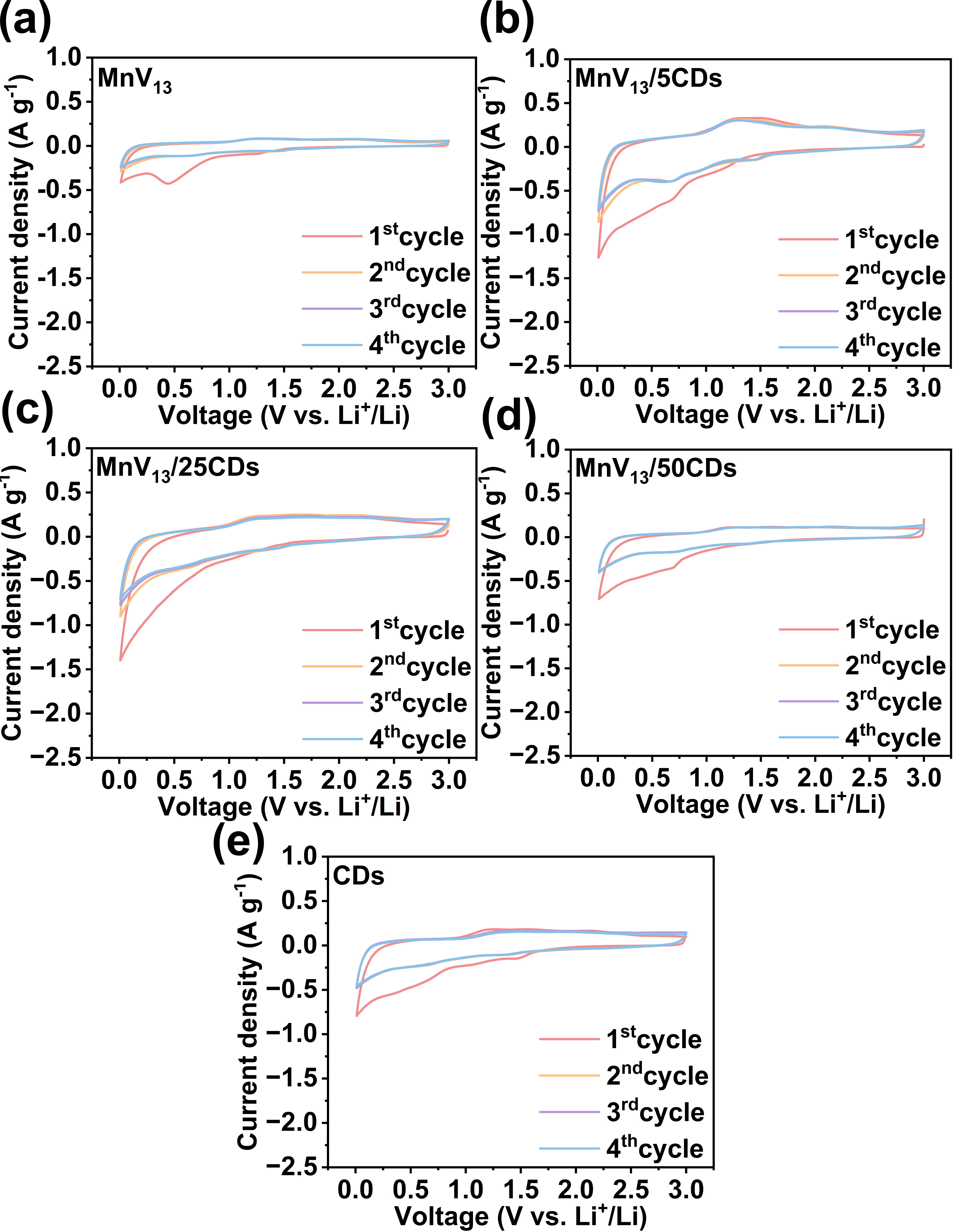


**Figure S6**. First four CV cycles of MnV_13_/*x*CDs as well as bare MnV_13_ and CDs at 0.2 mV s^−1^ within 0.01−3.0 V.


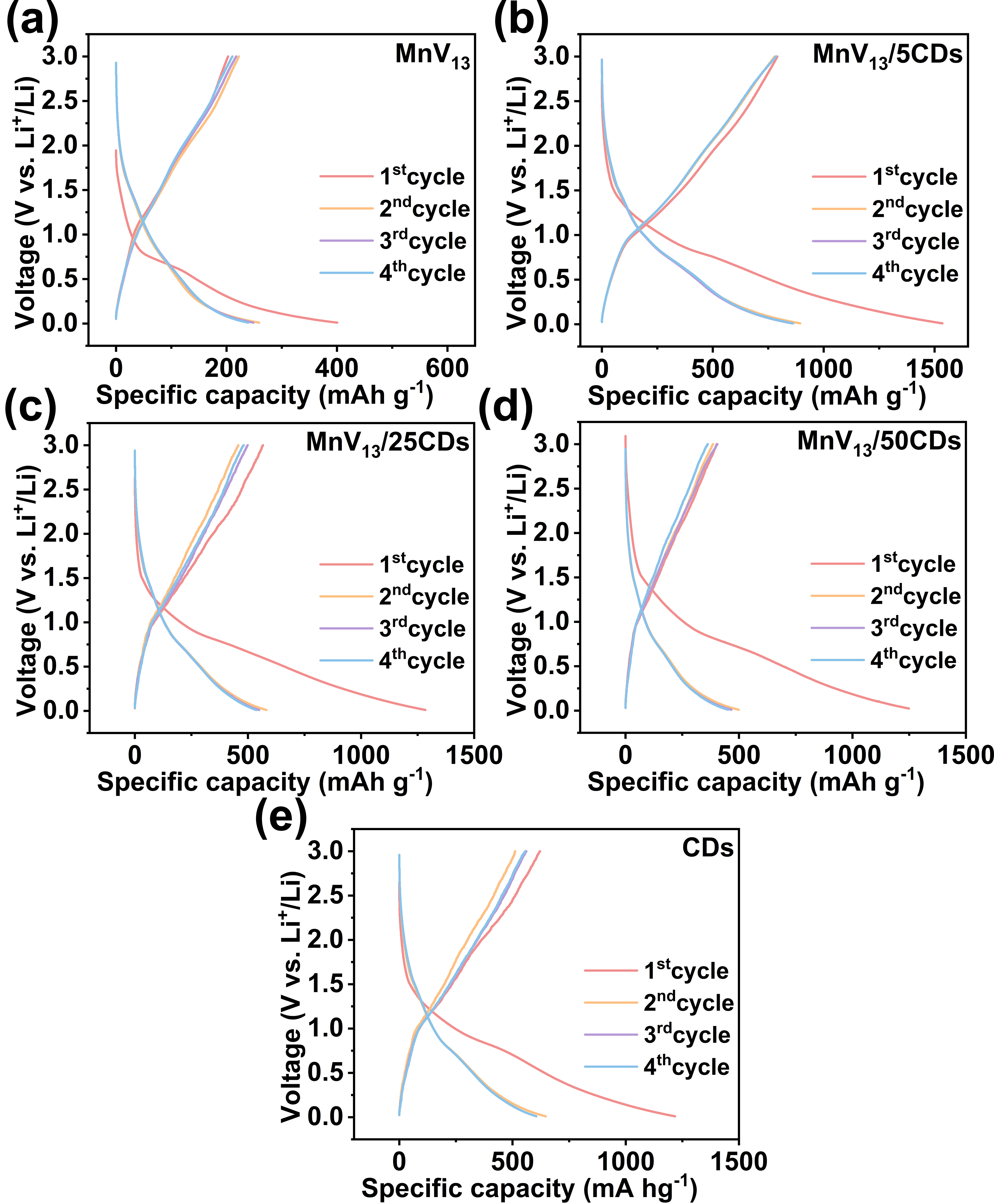


**Figure S7**. First four GCD cycles of MnV_13_/*x*CDs as well as bare MnV_13_ and CDs at a current density of 0.1 A g^−1^.


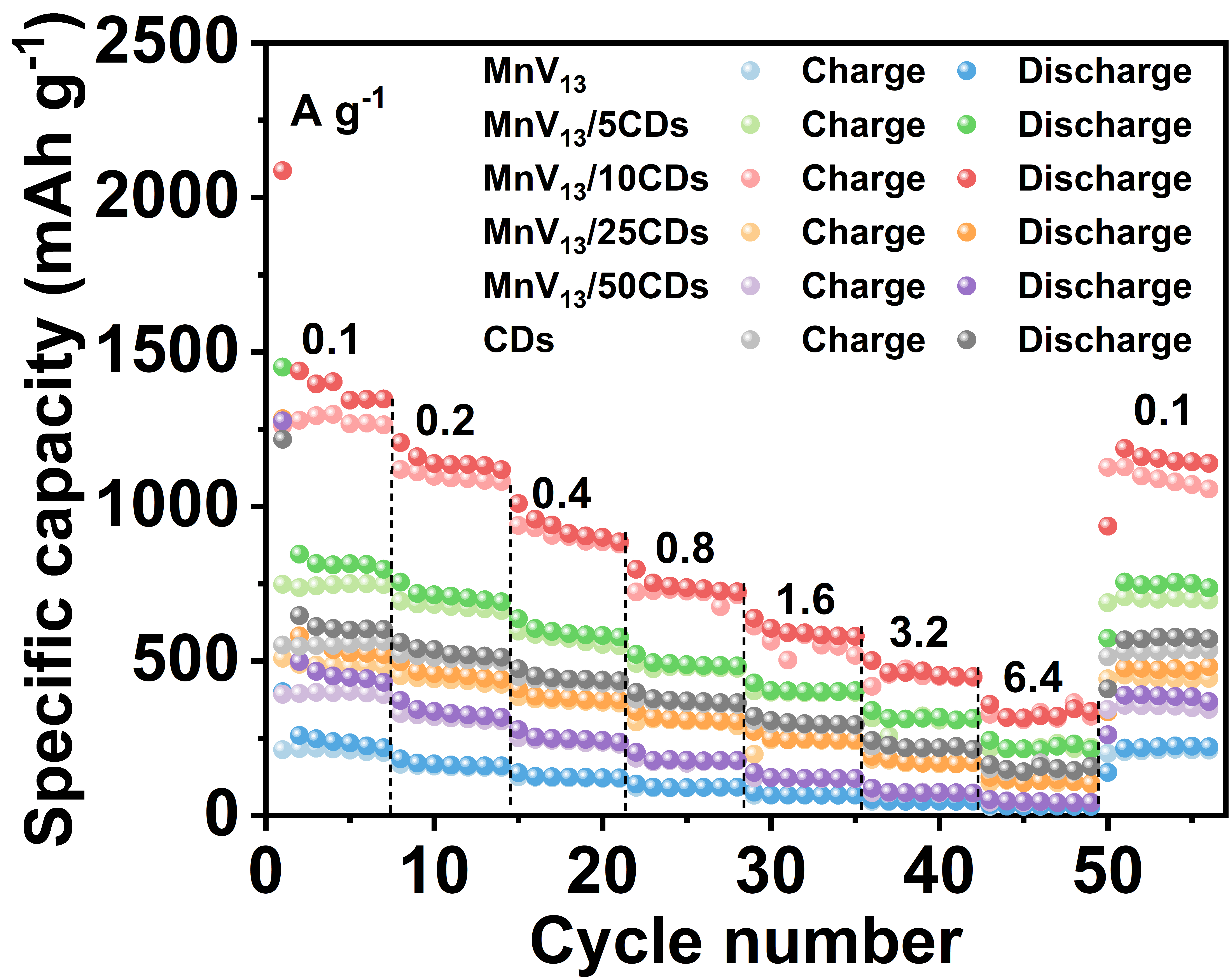


**Figure S8**. Rate performance of MnV_13_/*x*CDs as well as bare MnV_13_ and CDs.


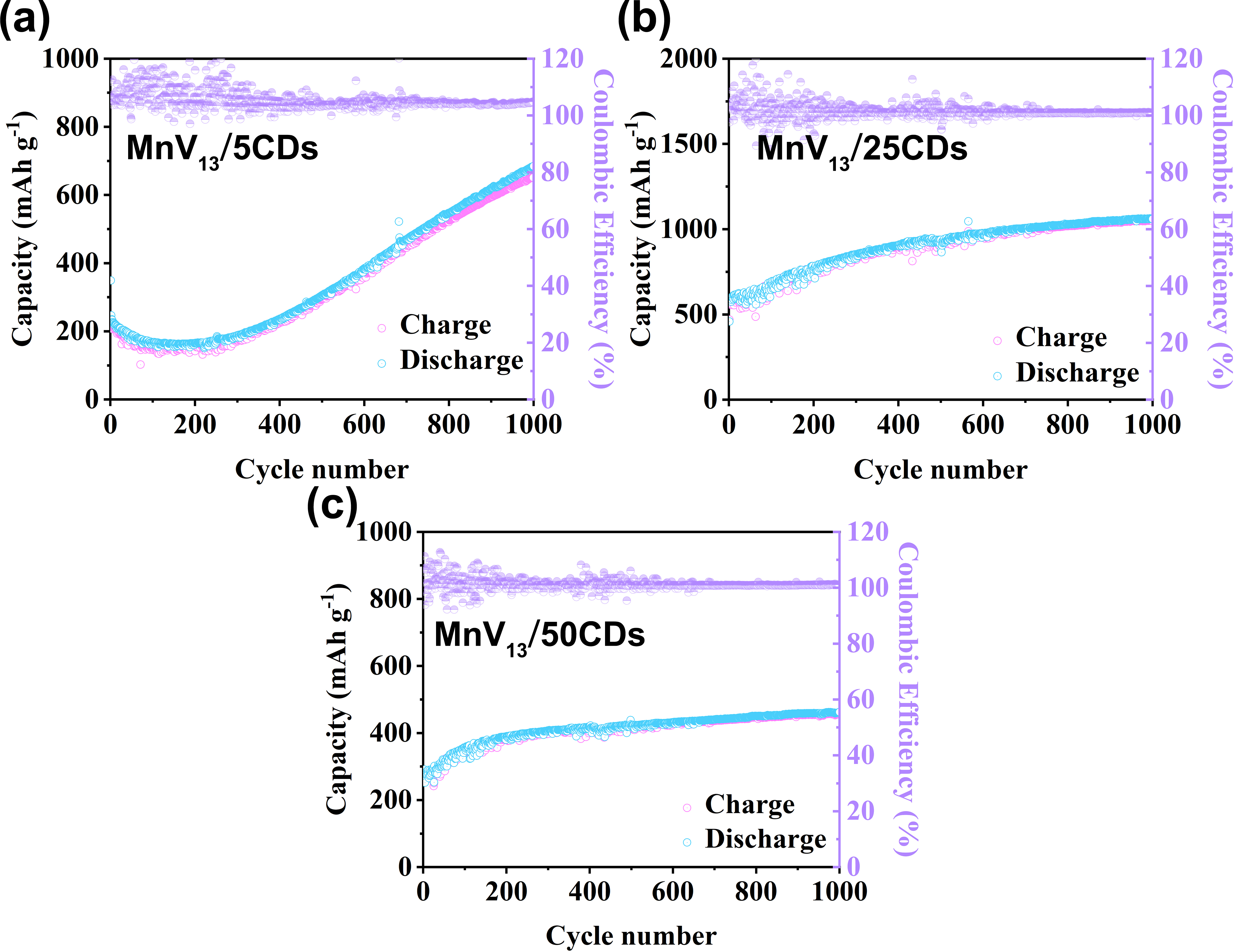


**Figure S9**. Cycling performance of MnV_13_/5CDs, MnV_13_/25CDs and MnV_13_/50CDs at 1 *C* for 1000 cycles.


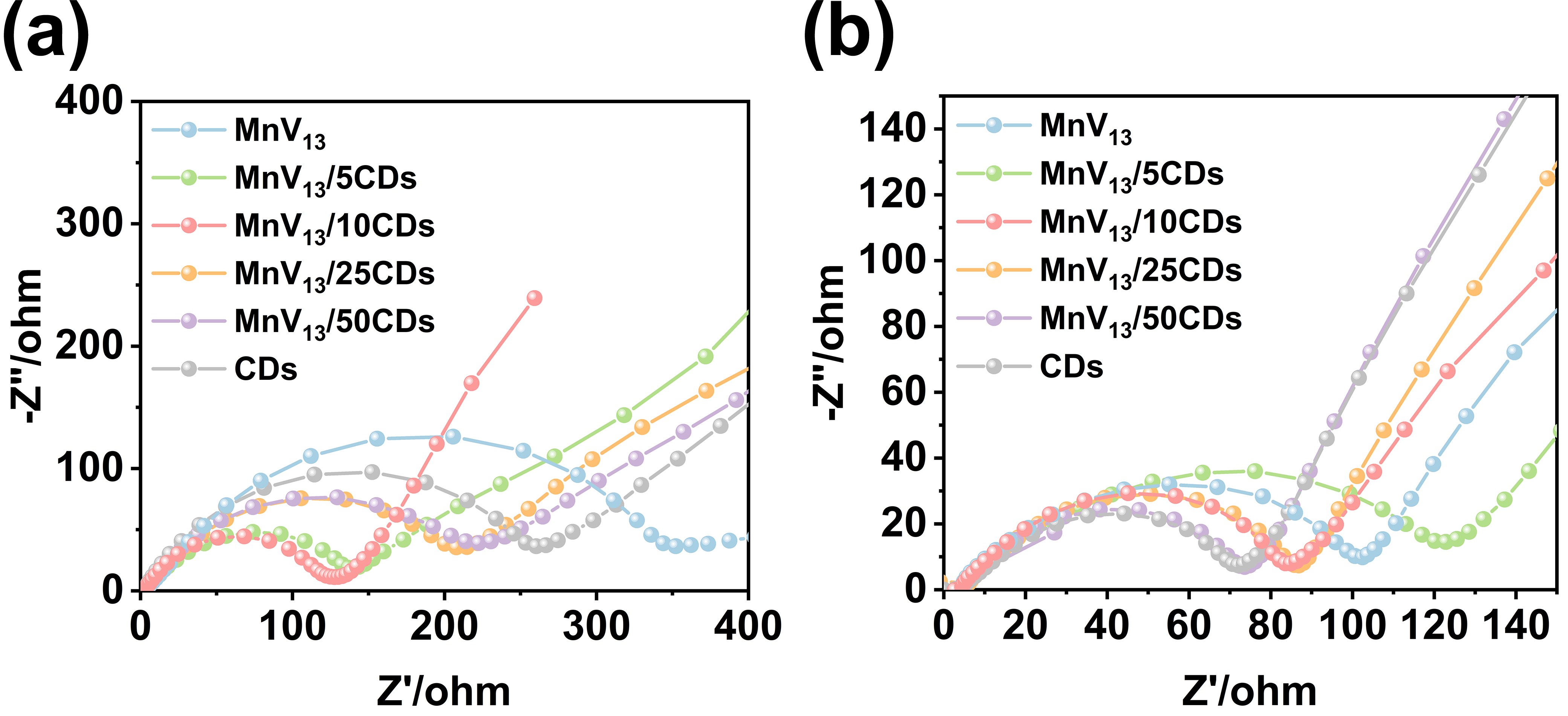


**Figure S10**. EIS measurements of MnV_13_/*x*CDs as well as bare MnV_13_ and CDs electrodes (a) before and (b) after 1000 cycles.


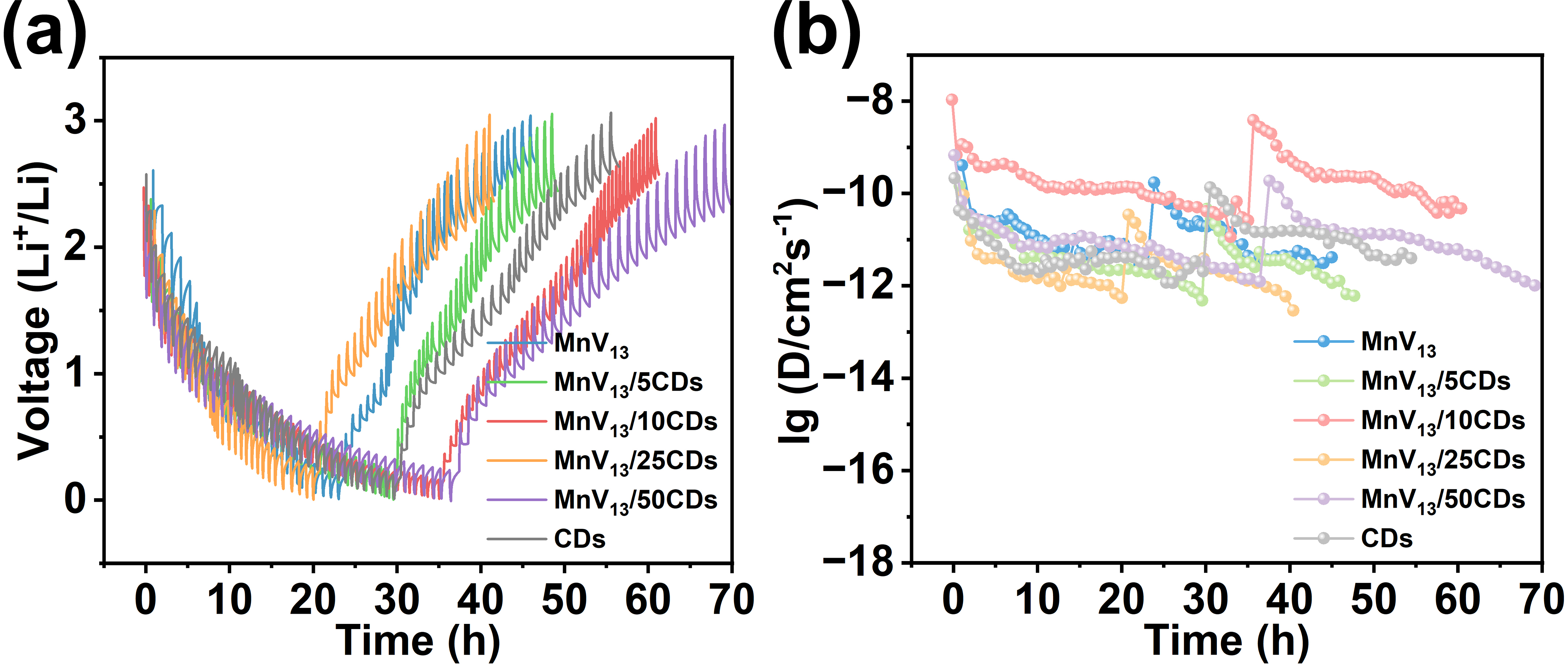


**Figure S11**. (a) GITT curves and (b) The calculated *D_Li_*^+^ values of the MnV_13_/*x*CDs as well as bare MnV_13_ and CDs.


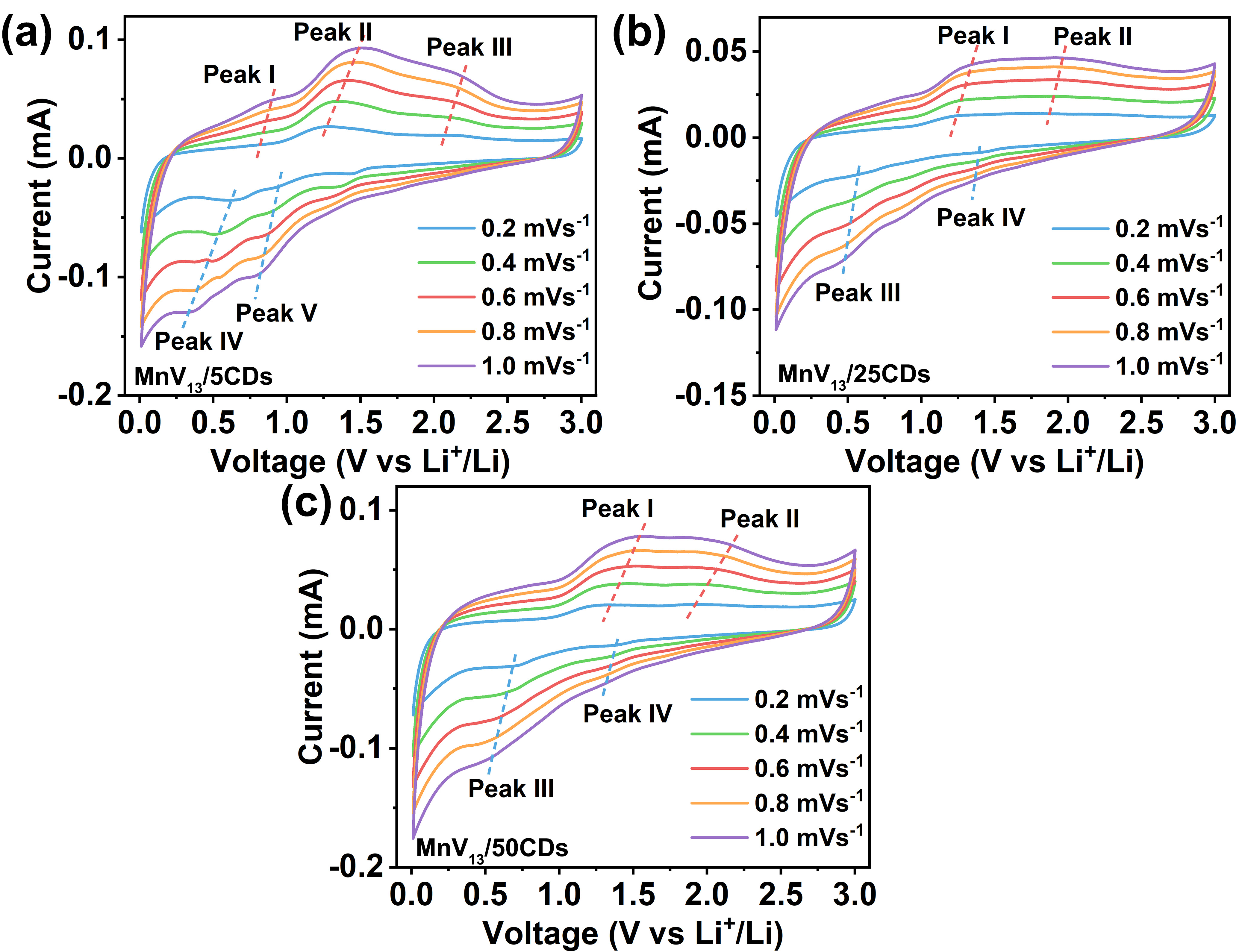


**Figure S12**. CV curves of (a) MnV_13_/5CDs, (b) MnV_13_/25CDs and (c) MnV_13_/50CDs at different scan rates.


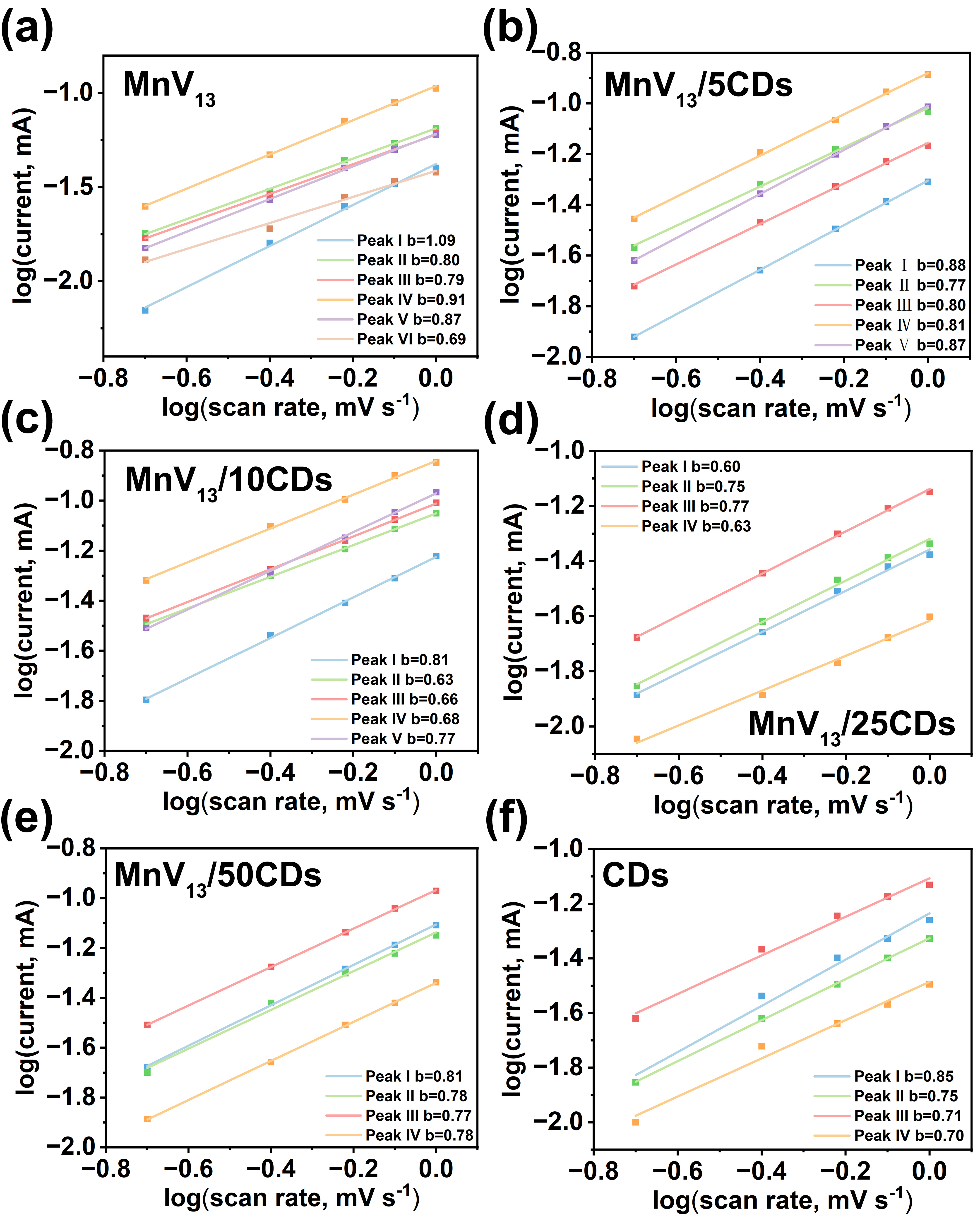


**Figure S13**. Linear fit of log(i) to log(𝜈) to determine the *b*-values of MnV_13_/*x*CDs as well as bare MnV_13_ and CDs.
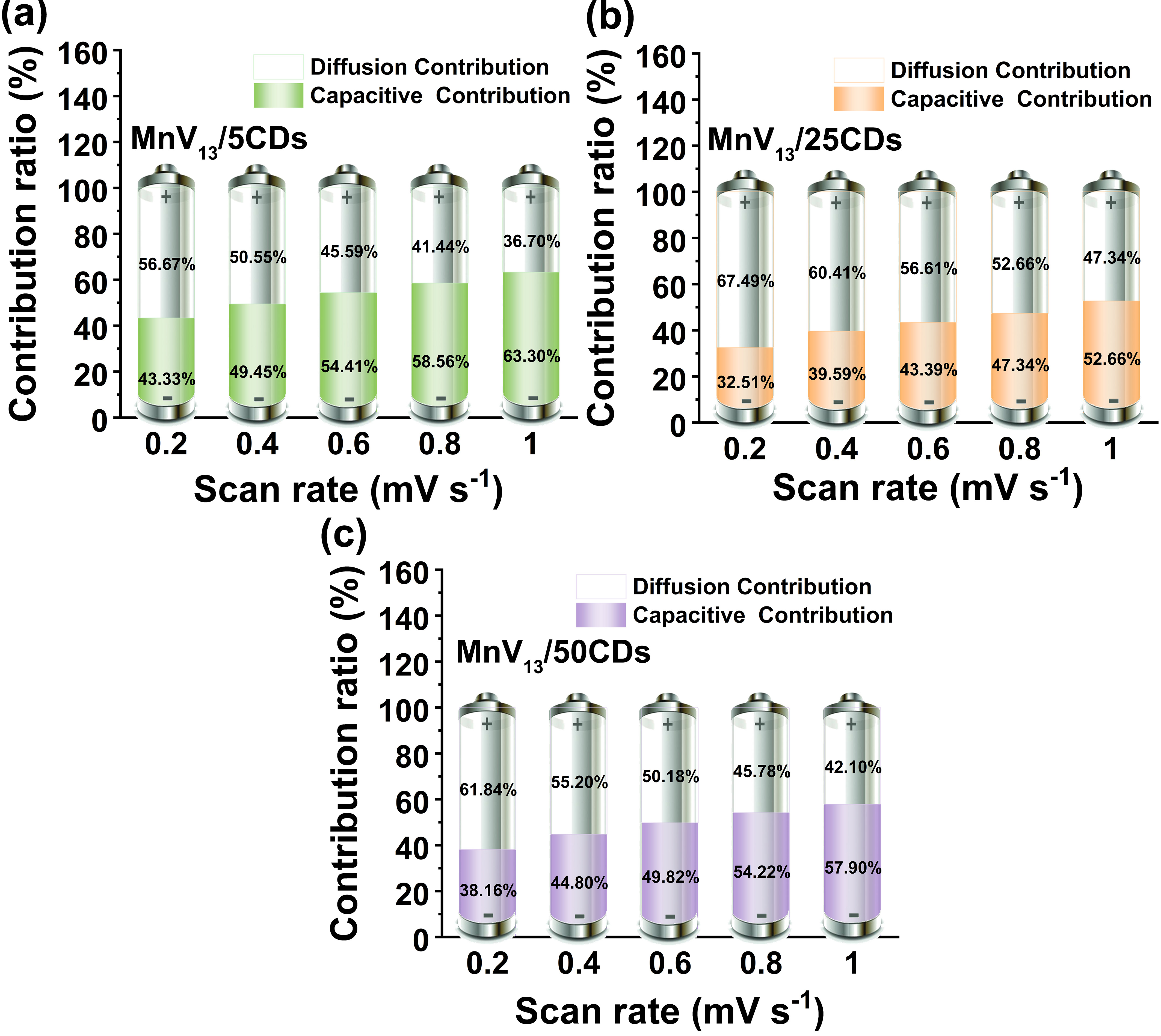


**Figure S14**. Diffusion contribution and Capacitive contribution of (a) MnV_13_/5CDs, (b) MnV_13_/25CDs and (c) MnV_13_/50CDs at various scan rates.

**Supplementary Tables**

**Table S1.** Comparison of the specific capacity of MnV_13_/*x*CDs as well as bare MnV_13_ and CDs.

| Electrode materials | Reversible Capacity  (mAh g^−1^) | Capacity retention (%) | Capacity maintained after cycling (%) |
| --- | --- | --- | --- |
| MnV_13_ | 253 | 3.2 | 100 |
| MnV_13_/5CDs | 903 | 27.7 | 84.6 |
| MnV_13_/10CDs | 1348 | 24.8 | 93.1 |
| MnV_13_/25CDs | 587 | 17.9 | 90.1 |
| MnV_13_/50CDs | 485 | 9.2 | 86.0 |
| CDs | 604 | 26.7 | 95.5 |

**Table S2.** Comparison of the specific capacity of MnV_13_/10CDs in this study with that of vanadium-based electrode materials and cluster materials recently reported for LIB applications.

| Electrode materials | Reversible Capacity  (mAh g^−1^) | Reference |
| --- | --- | --- |
| **CHVO** | **1175** | **[5]** |
| **VGSs** | **289** | **[6]** |
| **V_2_O_5_/graphene** | **361** | **[7]** |
| **V_2_O_5_/graphene** | **438** | **[8]** |
| **2DV_2_O_5_@CNS** | **860** | **[9]** |
| **gem-V_2_O_5_** | **131.9** | **[10]** |
| **VNF-C-120** | **184** | **[11]** |
| **CNT/V_2_O_5_** | **240** | **[12]** |
| **(NH_4_)_7_[MnV_13_O_38_]** | **234** | **[13]** |
| **NENU-507** | **640** | **[14]** |
| **K_5.72_H_3.28_[PV_14_O_42_]** | **230** | **[15]** |
| **K_7_NiV_13_O_38_** | **640** | **[16]** |
| **Li_7_[V_15_O_36_(CO_3_)]** | **170** | **[17]** |
| **NNU-11** | **750** | **[18]** |
| **CoZnCuNiFeZrCeO*x*-PMA** | **220** | **[19]** |
| **MnV_13_/10CDs** | **1348** | **This work** |

**References**:

[1] H. Li, X. He, Z. Kang, H. Huang, Y. Liu, J. Liu, S. Lian, C. H. A. Tsang, X. Yang, S.-T. Lee, *Angewandte Chemie International Edition* **2010**, *49*, 4430-4434.

[2] Flynn, C. M.; Pope, M. T. 1:13 Heteropolyvanadates of Manganese(IV) and Nickel(IV). *J. Am. Chem. Soc*. **1970**, 92, 85-90.

[3] a) P. E. Blöchl, *Physical Review B* **1994**, *50*, 17953-17979; b) G. Kresse, D. Joubert, *Physical Review B* **1999**, *59*, 1758-1775.

[4] G. Henkelman, B. P. Uberuaga, H. Jónsson, *The Journal of Chemical Physics* **2000**, *113*, 9901-9904.

[5] J. F. S. Fernando, D. P. Siriwardena, K. L. Firestein, C. Zhang, J. E. von Treifeldt, C.-E. M. Lewis, T. Wang, D. P. Dubal, D. V. Golberg, J. Mater. Chem. A. 2020, 8, 13183-13196.

[6] M. Yan, F. Wang, C. Han, X. Ma, X. Xu, Q. An, L. Xu, C. Niu, Y. Zhao, X. Tian, P. Hu, H. Wu, L. Mai, *J. Am. Chem. Soc.* **2013**, *135*, 18176-18182.

[7] F. Su, F. Xing, X. Wang, F. Liu, L. Zhang, Z.-S. Wu, *Energy Environ. Sci.* **2023**, *16*, 222-230.

[8] Q. Liu, Z.-F. Li, Y. Liu, H. Zhang, Y. Ren, C.-J. Sun, W. Lu, Y. Zhou, L. Stanciu, E. A. Stach, J. Xie, *Nat. Commun.* **2015**, *6*, 6127.

[9] X. Wang, W. Jia, L. Wang, Y. Huang, Y. Guo, Y. Sun, D. Jia, W. Pang, Z. Guo, X. Tang, *J. Mater. Chem. A.* **2016**, *4*, 13907-13915.

[10] M. Wilhelm, R. Adam, A. Bhardwaj, I. Neumann, S. H. Cho, Y. Yamada, T. Sekino, J. Tao, Z. Hong, T. Fischer, S. Mathur, *Adv. Eng. Mater.* **2023**, *25*, 2200765

[11] X. Jia, Z. Chen, A. Suwarnasarn, L. Rice, X. Wang, H. Sohn, Q. Zhang, B. M. Wu, F. Wei, Y. Lu, *Energy Environ. Sci.* **2012**, *5*, 6845-6849.

[12] R. C. McNulty, K. Penston, S. S. Amin, S. Stal, J. Y. Lee, M. Samperi, L. Pérez-García, J. M. Cameron, L. R. Johnson, D. B. Amabilino, G. N. Newton, *Angew. Chem., Int. Ed.* **2023**, *62*, e202216066

[13] W.-l. Li, E.-f. Ni, X.-h. Li, H.-j. Guo, *Trans. Nonferrous Met. Soc. China* **2016**, *26*, 2372-2379.

[14] Y.-Y. Wang, M. Zhang, S.-L. Li, S.-R. Zhang, W. Xie, J.-S. Qin, Z.-M. Su, Y.-Q. Lan, *Chem. Commun. (Cambridge, U. K.)* **2017**, *53*, 5204-5207.

[15] S. Uematsu, Z. Quan, Y. Suganuma, N. Sonoyama, *J. Power Sources* **2012**, *217*, 13-20.

[16] E. Ni, S. Uematsu, Z. Quan, N. Sonoyama, *J. Nanopart. Res.* **2013**, *15*, 1732.

[17] J.-J. Chen, M. D. Symes, S.-C. Fan, M.-S. Zheng, H. N. Miras, Q.-F. Dong, L. Cronin, *Adv. Mater.* **2015**, *27*, 4649-4654.

[18] Q. Huang, T. Wei, M. Zhang, L.-Z. Dong, A. M. Zhang, S.-L. Li, W.-J. Liu, J. Liu, Y.-Q. Lan, *J. Mater. Chem. A.* **2017**, *5*, 8477-8483.

[19] J. Liu, Y. Li, Z. Chen, N. Liu, L. Zheng, W. Shi, X. Wang, *J. Am. Chem. Soc.* **2022**, *144*, 23191-23197.
